# Assessment of Disrupted Brain Structural Connectome in Depressive Patients With Suicidal Ideation Using Generalized Q-Sampling MRI

**DOI:** 10.3389/fnhum.2021.711731

**Published:** 2021-08-27

**Authors:** Vincent Chin-Hung Chen, Chun-Ju Kao, Yuan-Hsiung Tsai, Man Teng Cheok, Roger S. McIntyre, Jun-Cheng Weng

**Affiliations:** ^1^School of Medicine, Chang Gung University, Taoyuan, Taiwan; ^2^Department of Psychiatry, Chang Gung Memorial Hospital, Chiayi, Taiwan; ^3^Department of Medical Imaging and Radiological Sciences, Bachelor Program in Artificial Intelligence, Chang Gung University, Taoyuan, Taiwan; ^4^Department of Diagnostic Radiology, Chang Gung Memorial Hospital, Chiayi, Taiwan; ^5^Graduate Institute of Biomedical Electronics and Bioinformatics, National Taiwan University, Taipei, Taiwan; ^6^Mood Disorder Psychopharmacology Unit, University Health Network, Department of Psychiatry, University of Toronto, Toronto, ON, Canada; ^7^Institute of Medical Science, University of Toronto, Toronto, ON, Canada; ^8^Departments of Psychiatry and Pharmacology, University of Toronto, Toronto, ON, Canada; ^9^Medical Imaging Research Center, Institute for Radiological Research, Chang Gung University and Chang Gung Memorial Hospital at Linkou, Taoyuan, Taiwan

**Keywords:** suicidal ideation, generalized q-sampling imaging, graph theoretical analysis, network-based statistical analysis, magnetic resonance imaging

## Abstract

Suicide is one of the leading causes of mortality worldwide. Various factors could lead to suicidal ideation (SI), while depression is the predominant cause among all mental disorders. Studies have shown that alterations in brain structures and networks may be highly associated with suicidality. This study investigated both neurological structural variations and network alterations in depressed patients with suicidal ideation by using generalized q-sampling imaging (GQI) and Graph Theoretical Analysis (GTA). This study recruited 155 participants and divided them into three groups: 44 depressed patients with suicidal ideation (SI+; 20 males and 24 females with mean age = 42, SD = 12), 56 depressed patients without suicidal ideation (Depressed; 24 males and 32 females with mean age = 45, SD = 11) and 55 healthy controls (HC; nine males and 46 females with mean age = 39, SD = 11). Both the generalized fractional anisotropy (GFA) and normalized quantitative anisotropy (NQA) values were evaluated in a voxel-based statistical analysis by GQI. We analyzed different topological parameters in the graph theoretical analysis and the subnetwork interconnections in the Network-based Statistical (NBS) analysis. In the voxel-based statistical analysis, both the GFA and NQA values in the SI+ group were generally lower than those in the Depressed and HC groups in the corpus callosum and cingulate gyrus. Furthermore, we found that the SI+ group demonstrated higher global integration and lower local segregation among the three groups of participants. In the network-based statistical analysis, we discovered that the SI+ group had stronger connections of subnetworks in the frontal lobe than the HC group. We found significant structural differences in depressed patients with suicidal ideation compared to depressed patients without suicidal ideation and healthy controls and we also found several network alterations among these groups of participants, which indicated that white matter integrity and network alterations are associated with patients with depression as well as suicidal ideation.

## Introduction

Suicide is one of the critical challenges for healthcare systems worldwide. More than 800,000 people die from suicide every year worldwide; moreover, suicide has become one of the leading causes of death for younger generations (Bilsen, 2018). Previous studies have indicated that various factors could lead to suicidal ideation, such as behaviors, including irregular impulse control, sociopsychological circumstances, a tendency to intense psychological pain, and a history of medical conditions (Joiner et al., 2005; Pompili et al., 2015). However, mental disorders are considered to be the predominant factor in suicidal ideation, with depression disorders as the main type of mental disorder linked to suicide ideation (Hawton et al., 2013). The World Health Organization (WHO) has ranked depressive disorder as one of the leading causes of mortality worldwide, and it is highly recurrent and prevalent and is associated with functional disability and morbidity (Richards, 2011; Murray et al., 2012). Depressed patients always encounter inconveniences in their lives due to their impairment of brain functions, including executive function, memory, and emotion processing (Mcintyre et al., 2013). Due to the seriousness and ubiquity of depression and suicidal ideation, numerous studies have been performed on depression in the fields of neuroimaging, neurobiology, psychiatry, animal testing and so on (Blazer, 2003; Dean and Keshavan, 2017; Liu et al., 2017; Planchez et al., 2019).

Previous studies have mainly focused on biological differences as well as physical behaviors instead of alterations in brain networks (Sotelo et al., 2014; Cuijpers et al., 2019). Nevertheless, brain network alterations are critical factors for depressed patients with suicidal ideation and they may be linked to suicide risks (Bani-Fatemi et al., 2018). In recent years, diverse medical imaging studies have been performed on brain structures and networks. Diffusion MRI, especially diffusion tensor imaging (DTI), has been broadly applied to numerous studies in the field of mental disorders (Blood et al., 2010; Thomason and Thompson, 2011; Cheng et al., 2014; Ota et al., 2015). This method is well known for its noninvasive features, which can map the orientations of neural fibers and also extract quantitative parameters. However, DTI is also known for its limitations in identifying crossing neural fibers (Yeh et al., 2010). Hence, we performed a different approach called generalized q-sampling imaging (GQI), which can reconstruct more precise and consistent images of complex oriented fibers than DTI (Yeh et al., 2010; Jin et al., 2019). Previous research also indicated that GQI demonstrates good sensitivity and specificity for the assessment of white matter connectivity (Yeh et al., 2010). For the clinical application of psychiatric disorders, a previous study also performed GQI for the investigation of major depressive disorder (Chen et al., 2016).

There has been a spurt of research on network modeling of brain connectivity, and graph theoretical analysis is growing rapidly in the field of neuroimaging (Bassett and Bullmore, 2009; He and Evans, 2010). Graph theory defines the connections of regions by illustrating pairwise interconnections called “edges” and “nodes” (Sporns, 2018). In our study, we defined nodes as brain regions and edges as structural interconnections. Since the brain is complicated with both segregated and integrated functions, graph theoretical analysis can be applied to evaluate network changes in the brain. The networks of the brain are known to specifically correspond with topological properties called “small-worldness” (Watts and Strogatz, 1998). Small-world network models could provide a versatile and powerful approach to understanding the structure and function of human brain systems (Bassett and Bullmore, 2006). Neuropsychiatric disorders are considered to be dysconnectivity syndromes, and graph theory has previously been used to quantify the variations in functional and structural network properties, which can be used to explore various disorders (Bullmore and Sporns, 2009; Nakamura et al., 2009; Kim et al., 2019). A previous DTI study found that depression is correlated with complex brain networks by network controllability analysis, yet the chosen partitions of brain modularity might bias its result (Kenett et al., 2018). Hence, we adopted graph theoretical analysis to investigate network alternations.

Previous noninvasive imaging studies showed replicated results of functional and structural alterations in people with suicidal ideation as well as depressive disorder and indicated various discoveries (Gong and He, 2015; Myung et al., 2016). Studies had indicated that several brain regions were highly associated with mental disorders, especially the corpus callosum and the cingulate (Janiri et al., 2020; Schmaal et al., 2020). Moreover, mental disorders such as mood and anxiety disorders were greatly associated with suicide (Henriksson et al., 1993; Harris and Barraclough, 1997). In this study, we performed an improved imaging approach with better specificity and sensitivity as well as a higher accuracy of fiber tracking and presupposed that suicidal ideation is significantly related to variations in particular brain regions. We also performed graph theoretical analysis to investigate the potential alteration of brain networks. Our study focused on investigating the variation of specific brain neurological structures and the alteration of brain network connectome among three groups of individuals, including depressed patients with suicidal ideation (SI+), depressed patients without suicidal ideation (Depressed), and healthy controls (HC) by use of extensive GQI and graph theoretical analysis.

## Materials and Methods

### Participants

In this cross-sectional study, we recruited a total of 155 participants from the Department of Psychiatry at Chiayi Chang Gung Hospital. We distributed the participants into three groups: 44 depressed patients with suicidal ideation (SI), 56 depressed patients without suicidal ideation (Depressed) and 55 healthy controls (HC). Our study was approved by the Institutional Review Board of the Chang Gung Memorial Hospital, Chiayi, Taiwan (No. 201901771B0, 201602027B0, 104-9337B). Patient assessments were confirmed on the basis of a structured interview with the Beck suicide intent scale and a Mini-International Neuropsychiatric Interview (MINI) by research nurses, along with diagnoses by psychiatrists. If one of the approaches revealed that the patient had the ideation of suicide, then he or she would be classified as the SI+ group. The average Beck’s SIS score of the SI+ group was 11.6 and the average Beck’s SIS score of the Depressed group was 1.9. The average illness duration of the participants in the Depressed group was approximately 4.5 years and the time window for SI to occur before scanning was 1 month. The inclusion criteria of the participants were as follows: right handed, more than 20 years old, and willing to sign the informed consent form. The exclusion criteria of the participants were as follows: history of suicide attempt, previously diagnosed with bipolar disorder, dissociation, schizophrenia, substance abuse or other mental disorder, a history of severe brain damage, pregnancy, illiteracy, and MRI contraindications. Sufficient communication was performed to ensure that every participant had clearly understood the guidelines and procedures. Participants who felt uncomfortable during the process were excluded from the study. The patients/participants provided their written informed consent to participate in this study.

### Diffusion MRI Data Acquisition

Participants in this study were scanned by a 3 T MRI imaging system (Verio, SIEMENS, Germany) at Chiayi Chang Gung Memorial Hospital. Single-shot and diffusion-weighted spin echo-planar imaging sequences were performed for diffusion imaging. The acquisition parameters of the images were as follows: repetition time (TR)/echo time (TE) = 8943/115 ms, matrix size = 128 × 128, slice thickness = 4 mm, field of view (FOV) = 250 × 250 mm^2^, voxel size = 3.4 × 3.4 × 4 mm^3^, number of excitations = 1, b-values = 0, 1,000, 1,500 and 2,000 s/mm^2^ in a total of 193 noncollinear directions. The total acquisition time of each participant was approximately 30 min.

### Generalized Q-Sampling Imaging (GQI)

A unique reconstruction method named generalized q-sampling imaging (GQI) is an approach that can provide added information with various quantitative indices, such as generalized fractional anisotropy (GFA), quantitative anisotropy (QA), normalized quantitative anisotropy (NQA), and the isotropic value of the orientation distribution function (ISO; Yeh et al., 2010). GFA is defined as the measurement of neural anisotropy, which is calculated by orientation distribution function (ODF) and is highly associated with fractional anisotropy (FA). The value of GFA would decrease in some fiber crossing or partial volume regions as well as the voxels contain cerebrospinal fluid; NQA is the normalized QA; QA indicates the number of anisotropic spins that diffuse along fiber orientations. It is calculated by the directions of spin distribution function (SDF), which is highly associated with the directions of neural fibers. In addition, QA and NQA can be less interfere by partial volume effect compared to FA and GFA, since they are correlated to spin density. ISO represents the background isotropic diffusion (Yeh et al., 2010). We specifically focused on the value of both GFA and NQA to investigate white matter integrity in this study. Furthermore, participants’ age, gender, years of education, Hamilton Rating Scale for Depression (HAM-D) score without suicidal factors, and Hospital Anxiety and Depression Scale (HADS) score (part of anxiety) were five covariates that were considered to be no interest.

### Diffusion Imaging Preprocessing

The first step is to ensure the quality of the diffusion imaging. We performed eddy current correction by FSL (FMRIB Software Library), which could minimize characteristic eddy current artifacts caused by echo-planar imaging sequences. Next, we normalized the shapes and sizes of all imaging data by using Statistical Parametric Mapping 8 (SPM8; Wellcome Department of Cognitive Neurology, London, UK). When operating the step of normalization in SPM8, we normalized the images of each b-value with DARTEL respectively to ensure that further statistical analysis could have more precise results. We reconstructed two GQI indices (GFA and NQA) by applying DSI Studio (Yeh et al., 2010).

### Voxel-Based Statistical Analysis

We used SPM8 to perform ANCOVA to assess the differences within the three groups. Then, we performed two-sample *t*-tests for further investigation to evaluate the values of GFA and NQA within different regions of the brain among the three groups by using SPM8. In our voxel-based statistical analysis, participants’ age, gender, years of education, Hamilton Rating Scale for Depression (HAM-D) score without suicidal factors, and Hospital Anxiety and Depression Scale (HADS) score (part of anxiety) were five covariates that were considered to be no interest. Additionally, a false discovery rate (FDR)-corrected *P*-value of <0.05 was considered to be statistically significant.

### Graph Theoretical Analysis (GTA)

For network measurement, we extracted the GQI datum, which we analyzed, to perform graph theoretical analysis (GTA) using the Graph Analysis Toolbox (GAT, Stanford University School of Medicine, Stanford, CA, USA; Hosseini et al., 2012). We divided the brain into 90 regions in the Montreal Neurological Institute (MNI) space on the basis of the Automated Anatomical Labeling (AAL) atlas and reestablished neural fiber pathways using fiber assignment by continuous tracking (FACT) by using DSI Studio (Tzourio-Mazoyer et al., 2002). In addition, we defined every brain region as a “node”, and the connectivity between each node was defined as an “edge” using FACT and AAL templates (Behzadi et al., 2007). The edge was defined as the connection of neural fibers, which was calculated by the equation of numbers of connected fibers/average length of connected fibers x NQA. The number of edges connected to each pair of regions was determined and this resulted in the 90 × 90 connectivity matrix for each participant (Kesler et al., 2015). The “degree” of each node can illustrate the distribution of the edges in the brain networks because it represents the number of edges that connect to the rest of the network, and the “density” of the network represents the fraction of present connections to all possible connections (Bullmore and Sporns, 2009). In GTA, we can obtain various topological parameters that demonstrate global integration or local segregation of complex brain networks. The topological parameters that we acquired included global efficiency, normalized shortest path length (λ), characteristic path length, normalized clustering coefficient (γ), and the small-worldness index. The small-worldness index is defined as the normalized clustering coefficient (γ) divided by the normalized shortest path length (λ). In addition, all participants’ age, gender, years of education, Hamilton Rating Scale for Depression (HAM-D) score without suicidal factors, and Hospital Anxiety and Depression Scale (HADS) score (part of anxiety) were considered to be five covariates as no interest. The network density range was calculated between 0.08 and 0.25 with an accretion of 0.01 to conduct topological parameters, while the area under the curve (AUC) of topological parameters among selected ranges was compared between groups to evaluate significant differences.

### Network-Based Statistical (NBS) Analysis

Network-based statistical (NBS) analysis is based on a statistically parametric map with cluster-based threshold links that can be used to demonstrate group comparisons and identify differences in connected brain networks (Zalesky et al., 2010). Regional subnetworks were evaluated between groups by using two-sample *t*-tests. To investigate the significance of the linked edges, the distribution of the edge number was acquired empirically by nonparametric permutation. Then, the significant subnetworks that were constructed by NBS were visualized by BrainNet viewer (The MathWorks Inc., Natick, MA, USA; Xia et al., 2013).

## Results

### Demographic Characteristics

Table 1 illustrates the demographic characteristics of the participants. Group comparisons were performed to assess the notable differences between groups. Additionally, five covariates, gender, age, years of education, Hamilton Rating Scale for Depression (HAM-D; subtracting the factor of suicide), and the anxiety score of the Hospital Anxiety and Depression Scale (HADS), were considered to modify the impact for subsequent analyses.

**Table 1:** Demographic characteristics.

Characteristics	SI+ (*n* = 44)		Depressed (*n* = 56)		HC (*n* = 55)		ANCOVA	SI+ vs. HC	SI+ vs. Depressed	Depressed vs. HC
	Mean of counts	SD	Mean of counts	SD	Mean of counts	SD	*P*-value			
Gender (M/F)	20/24	N/A	24/32	N/A	9/46	N/A	N/A	0.002	0.798	0.002
Age	41.59	11.96	45.48	10.53	39.4	10.70	0.015	0.345	0.092	0.003
Range of age	20–60	N/A	20–60	N/A	20–57	N/A	N/A	N/A	N/A	N/A
Years of education	12.66	3.21	13.25	2.82	14.25	2.98	0.029	0.013	0.338	0.073
HAM-D	17.59	5.85	14.89	6.54	3.93	5.51	<0.001	<0.001	0.032	<0.001
HAM-D (without suicidal factor)	16.11	5.65	13.34	6.75	3.82	5.31	<0.001	<0.001	0.028	<0.001
Anxiety of HADS	12.02	3.92	7.95	4.46	4.31	3.63	<0.001	<0.001	<0.001	<0.001
Depression of HADS	12.68	4.31	7.14	4.57	3.30	3.22	<0.001	<0.001	<0.001	<0.001

*Abbreviation: SI+, Depressed patients with suicidal ideation; Depressed, Depressed patients without suicidal ideation; HC, Healthy controls; HAM-D, Hamilton Rating Scale for Depression; HADS, Hospital Anxiety and Depression Scale; SD, Standard deviation*.

### Voxel-Based Statistical Analysis

**Figure 1:**
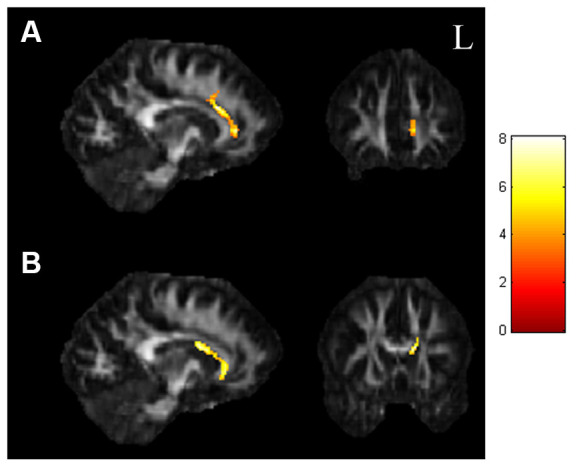
ANCOVA results of GFA and NQA. **(A)** The GFA value showed significant differences in the corpus callosum and anterior cingulate among the three groups. **(B)** The NQA value showed significant differences in the corpus callosum and anterior cingulate among the three groups (*p* < 0.05, cluster size >100, color bar: F scores). GFA, generalized fractional anisotropy; NQA, normalized quantitative anisotropy.

**Figure 2:**
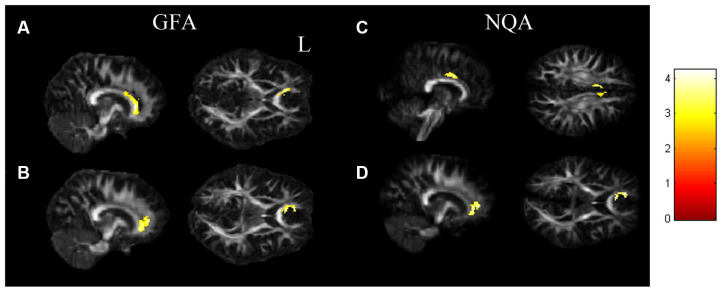
The *t*-test results. **(A)** The SI+ group showed significantly lower GFA values in the corpus callosum, anterior cingulate than the HC group. **(B)** Compared to the Depressed group, the SI+ group showed significantly lower GFA in the corpus callosum, anterior cingulate. **(C)** The SI+ group demonstrated notably lower NQA values in the anterior cingulate than the HC group. **(D)** Compared to the Depressed group, the SI+ group demonstrated notably lower NQA in the corpus callosum, anterior cingulate (*p* < 0.05, cluster size > 100, color bar: *t* scores).

In the ANCOVA results shown in Figure 1 and Supplementary Table 1, the GFA value demonstrated notable differences in the corpus callosum and anterior cingulate (Figure 1A). The value of NQA demonstrated notable differences in the corpus callosum and anterior cingulate (Figure 1B). In the comparisons of two-sample *t*-tests (Figure 2 and Supplementary Table 2), the GFA of the SI+ group was significantly lower in the corpus callosum and anterior cingulate than in the HC group (Figure 2A). GFA of the SI+ group was notably lower in the corpus callosum and anterior cingulate than that of the Depressed group (Figure 2B). Regarding the value of the NQA, the NQA of the SI+ group was remarkably lower in the anterior cingulate than that of the HC group (Figure 2C). The NQA of the SI+ group was significantly lower in the corpus callosum and anterior cingulate than that of the Depressed group (Figure 2D).

### Graph Theoretical Analysis

**Figure 3:**
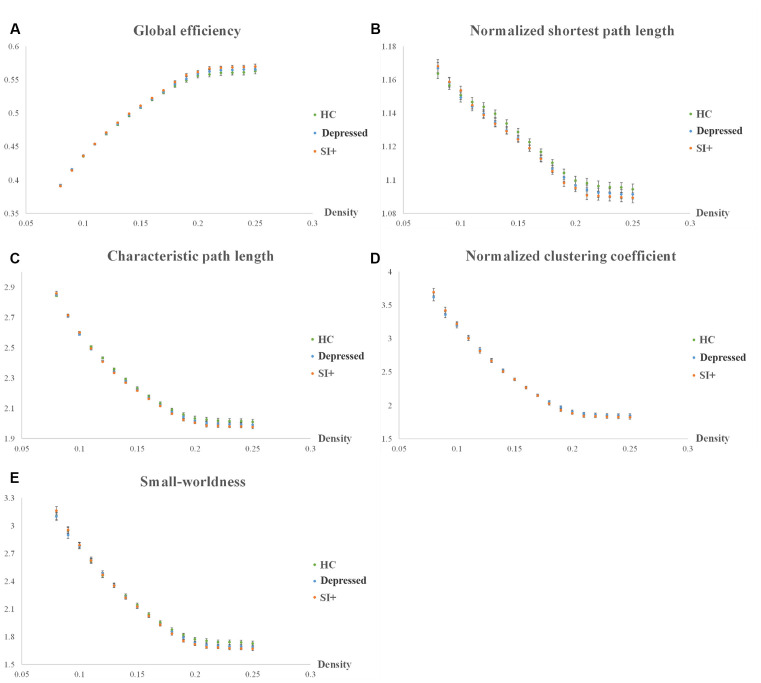
Topological parameters of graph theoretical analysis. Five topological parameters were investigated among the three groups (SI, Depressed, and HC): **(A)** global efficiency, **(B)** normalized shortest path length (λ), **(C)** characteristic path length, **(D)** normalized clustering coefficient (γ), and **(E)** small-worldness index.

The statistical results were not significant (*p* > 0.05) in the graph theoretical analysis; however, we did find remarkable tendencies in some of the topological parameters, such as global efficiency, normalized shortest path length (λ), characteristic path length, normalized clustering coefficient (γ), and the small-worldness index (Figure 3). We extracted and assessed the density from 0.08 to 0.25 by AUC analysis. The networks of all three groups remained constant when the density was beyond 0.25, and the networks oscillated when the density was beneath 0.08, which was not meaningful for the analysis of group comparisons. In the comparisons between groups, the SI+ group demonstrated the highest global efficiency (Figure 3A). It also demonstrated the shortest normalized shortest path length (λ), characteristic path length (Figures 3B,C), and the lowest normalized clustering coefficient (γ; Figure 3D). Our results showed that the small-worldness index was more than 1 in all three groups (Figure 3E).

### Network-Based Statistical (NBS) Analysis

**Figure 4:**
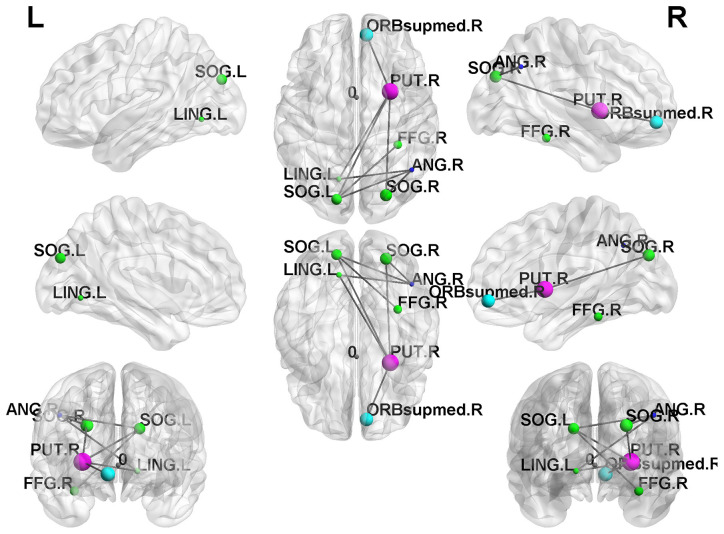
Network-based statistical (NBS) results. The SI+ group demonstrated significantly reduced subnetwork connections in the frontal lobe compared to the HC group (*p* < 0.05).

In the result of the NBS analysis, we found that the SI+ group retained significantly weaker interconnections of subnetworks in the frontal lobe than the HC group in the NBS analysis (Figure 4). However, we did not find significant network differences in the SI+ group compared to the Depressed group.

## Discussion

Our study explored microstructural and topological changes in depressed individuals with suicidal ideation compared to depressed individuals without suicidal ideation and healthy controls.

### Voxel-Based Statistical Analysis

In our study, we analyzed both GFA and NQA values of the three groups (SI, Depressed, and HC). These two parameters were reconstructed from the GQI, which represents the degree of myelination and the normalized value of anisotropy based on the measurement of restricted diffusion (Yeh et al., 2010). In our voxel-based analysis, we first performed ANCOVA to observe the brain regions that had alterations in GFA and NQA values among the SI+, Depressed, and HC groups. We found that some regions demonstrated significant results, including the corpus callosum and cingulate gyrus. According to previous studies, these brain regions are highly associated with mental disorders such as anxiety and mood disorders, especially the corpus callosum and the anterior cingulate (Janiri et al., 2020; Schmaal et al., 2020). Previous studies have emphasized that mental disorders are substantially associated with suicide (Henriksson et al., 1993; Harris and Barraclough, 1997). Moreover, we performed two-sample *t*-tests to make group comparisons. The results indicated that the SI+ group had lower GFA and NQA values than the Depressed and HC groups; that is, the white matter integrity of the SI+ group was worse than that of both the Depressed and HC groups. This result was consistent with previous studies (Graziano et al., 2019; Zhang et al., 2019). Our findings showed that brain regions including the corpus callosum and cingulate gyrus had notable neurological structural variations.

### Corpus Callosum

Our study indicated a huge reduction in white matter connectivity in the corpus callosum of the SI+ group. The corpus callosum is the largest white matter structure inside the human brain, connecting many cortical regions across both hemispheres, and it is related to various brain functions (Van der Knaap and Van der Ham, 2011). Previous studies have indicated that the corpus callosum plays an important role in emotional processing, working memory, and cognitive control in patients with major depressive disorders (Phan et al., 2002; Matsuo et al., 2007; Wagner et al., 2008). In addition, the corpus callosum is also highly related to executive function and emotion regulation (Balevich et al., 2015; Gifuni et al., 2017). Patients with impairment of the corpus callosum may lose various brain functions and suffer from psychiatric disorders, such as schizophrenia or bipolar disorder, with a higher risk of suicide (Balevich et al., 2015; Gifuni et al., 2017). The size of the corpus callosum is also related to emotional deficits and cognitive impairment, while such deficits can also be found in patients with suicidal behaviors (Cyprien et al., 2011). For neurological evaluations, a previous study showed that the corpus callosum had lower fractional anisotropy (FA) values in patients with suicidal ideation (Zhang et al., 2019), which was the same as our findings. Another study indicated that the integrity of the corpus callosum was associated with a suicide attempt history (Cyprien et al., 2016), which verified our results.

### Cingulate Gyrus

In the SI+ group, we found that the connectivity of the white matter decreased significantly in the cingulate gyrus compared to the Depressed and HC groups, especially the anterior section. From the perspective of anatomy, the cingulate is associated with many limbic and cortical structures, and it straddles the domains of both emotion and memory (Bubb et al., 2018; Rolls, 2019). The anterior part of the cingulate is primarily related to emotion and is sometimes called the “anterior emotional subsystem” (Vogt, 2019). Another neuroimaging study indicated that the cingulate gyrus is related to memory and emotion processing (Hadland et al., 2003). Therefore, impairment of the cingulate could cause unusual emotional responses and lead to psychiatric disorders. Previous fMRI studies showed that the cingulate of patients with anxiety and mood disorders had lower activation than in healthy controls, as did adolescents with suicide attempts and ideation (Pico-Perez et al., 2017; Harms et al., 2019). Regarding the white matter, suicidal brains were associated with a reduction in white matter volume (Bani-Fatemi et al., 2018; Huber et al., 2021). Reduced structural connectivity of the cingulate was also found in suicidal patients, which is consistent with our results (Schmaal et al., 2020). In addition, the anterior cingulate could have the potential for predicting the response to antidepressants in the clinic (Godlewska et al., 2018).

### Network Properties

Previous studies have demonstrated that depression, as well as suicidal ideation and attempts, may be associated with alterations in the brain network (Dusi et al., 2015; Bani-Fatemi et al., 2018; Li et al., 2018). In our graph theoretical analysis, we investigated five topological parameters: global efficiency, normalized shortest path length (λ), characteristic path length, normalized clustering coefficient (γ), and the small-worldness index. In the group comparison, we found that the SI+ group had different tendencies compared to the Depressed and HC groups. The SI+ group showed the highest global efficiency, which indicated that they had an increase in global integration in the brain network. The SI+ group also showed a notably shorter normalized shortest path length (λ) and characteristic path length than both the Depressed and HC groups. The normalized shortest path length (λ) defines the minimum step numbers to travel between two nodes, which provides insight into the connections between remote regions of the brain network (Gong and He, 2015). The characteristic path length describes the average length of the shortest path between all possible pairs of nodes in the network (Lin et al., 2018). As both lengths become shorter, the ability of global integration in the brain improves. Moreover, our results showed a reduction in the normalized clustering coefficient (γ) in the SI+ group, which indicated that the SI+ group had the worst local segregation since the normalized clustering coefficient (γ) is the normalization of the clustering coefficient that locally quantifies network interconnections. The small-worldness index is the ratio of the normalized clustering coefficient (γ) to the normalized shortest path length (λ).

Our results showed that the small-worldness indices of the SI+, Depressed, and HC groups were all greater than 1, which illustrated that small-world properties remained in all groups. “Small-world” is a term that describes brain networks that combine both high clustering and high efficiency, that is, regular and random topological properties, respectively (Latora and Marchiori, 2001). Small-world properties can also be regarded as being efficient in both local and global communication (Latora and Marchiori, 2001). Ideal complex brain networks have a small-world feature that is characterized by local clustering of connections between adjacent nodes and a short path length between distant node pairs; that is, a small-world property can support both segregated and integrated information processing of the brain (Bassett and Bullmore, 2006). Another characteristic of small-world networks is that they work economically, which minimizes the costs of wiring and supports high dynamical complexity, while they have resilience against certain types of brain damage (Bassett and Bullmore, 2006; Reijneveld et al., 2007).

In our network measurements, greater global consolidation accompanied by declined local cliques in the SI+ group illustrated that it was inclined toward a random network compared to the Depressed and HC groups, which is consistent with previous studies (Hwang et al., 2018). Random networks are those in which two given nodes are connected by one edge, which displays higher global integration and are less efficient since they spend too much energy in maintaining brain function. However, the brain networks of the SI+ group still retained their small-world properties (small-worldness index >1). In addition, the NBS analysis results indicated that the SI+ group had much fewer interconnections in the frontal lobe than the HC group. Various studies had mentioned that the frontal region of the brain is highly associated with depression as well as suicide. Previous research indicated that depressed patients with a suicide history might have abnormal frontothalamic loops, which is consistent with our findings (Jia et al., 2014).

We discovered that various brain regions showed a significant deterioration of white matter integrity, which was highly associated with network alterations. According to our study, we conjectured that the impairment of white matter connectivity might induce a compensatory mechanism that involves the remodeling of complex brain networks.

## Limitations and Future Directions

Some potential limitations are present in our study. For instance, our study only focused on adult individuals, with no juveniles. However, adolescents may demonstrate different results than adult individuals since adolescence and childhood are considered to be major periods during the maturation of the white matter (Paus et al., 2001). In addition, our study recruited 55 healthy controls, including nine males and 46 females. Although healthy individuals were not the main targets of investigation in this study, however, future studies could avoid recruiting participants with such gender differences.

In regard to depressive disorder and its associated impact, such as suicide, discoveries may be inconsistent across studies due to restricted statistical power related to insufficient sample sizes as well as disparate analytical methods and techniques (Van Velzen et al., 2020). Future studies could recruit larger sample sizes as well as different clinical populations globally, which could provide us with more robust findings (Button et al., 2013). Studies could also investigate more aspects of suicidal risks, such as hopelessness and rumination (Miranda and Nolen-Hoeksema, 2007). Furthermore, there is a consensus indicating that childhood trauma may be related to the development of depression (Negele et al., 2015); moreover, childhood abuse and peer bullying could form a prelude to suicidal ideation or behaviors in the future (Peyre et al., 2017; Lebowitz et al., 2019). Therefore, social experiences should also be investigated in future studies since advanced interventions for psychiatric disorders may reduce the burden of suicide and may be helpful for clinical diagnoses.

## Conclusion

This study investigated the variation of specific brain neurological structures and the alteration of the brain network connectome among three groups of individuals, including healthy controls and depressed patients with and without suicidal ideation. We found significant results that adequately indicated that both white matter integrity, as well as network alterations, are associated with patients with depression as well as suicidal ideation. More research could be performed in the future to gain further understanding, which could also have clinical applications.

## Data Availability Statement

The datasets presented in this article are not readily available because of the requirements of the study’s ethical approval and the requirements of data protection legislation. The dataset will only be made available on a restricted basis according to the data sharing policies at Chang Gung University and Chang Gung Memorial Hospital. Requests to access the datasets should be directed to Jun-Cheng Weng, jcweng@mail.cgu.edu.tw.

## Ethics Statement

The studies involving human participants were reviewed and approved by the Institutional Review Board of the Chang Gung Memorial Hospital, Chiayi, Taiwan (No. 201901771B0, 201602027B0, 104-9337B). The patients/participants provided their written informed consent to participate in this study.

## Author Contributions

J-CW, VC, C-JK, Y-HT, and MC conceived and were involved in study design, analysis, discussion, and interpretation of results. C-JK, J-CW, and RM drafted and refined the manuscript. All authors contributed to the article and approved the submitted version.

## Conflict of Interest

The authors declare that the research was conducted in the absence of any commercial or financial relationships that could be construed as a potential conflict of interest.

## Publisher’s Note

All claims expressed in this article are solely those of the authors and do not necessarily represent those of their affiliated organizations, or those of the publisher, the editors and the reviewers. Any product that may be evaluated in this article, or claim that may be made by its manufacturer, is not guaranteed or endorsed by the publisher.
