# Supplementary material for: Assessment of Disrupted Brain Structural Connectome in Depressive Patients With Suicidal Ideation Using Generalized Q-Sampling MRI
Source: Front Hum Neurosci. 2021 Aug 27;15:711731. doi: 10.3389/fnhum.2021.711731 (PMC8430248; doi:10.3389/fnhum.2021.711731)
Supplement: Supplementary file 1 [file Data_Sheet_1.docx]

| Table S1. Regions showing GFA and NQA values differences between depressed patients with suicidal ideation (SI), depressed patients without suicidal ideation (Depressed) and healthy controls (HC) by ANCOVA. | | | | |
| --- | --- | --- | --- | --- |
| Region | side | MNI coordinates (mm) | | |
|  |  | X | Y | Z |
| GFA |  |  |  |  |
| Corpus callosum and anterior cingulate | L/R | ±14 | 32 | 11 |
|  |  |  |  |  |
| NQA |  |  |  |  |
| Corpus callosum and anterior cingulate | L/R | ±14 | 11 | 17 |
| All coordinates are given in Montreal Neurological Institute (MNI) space. (p < 0.05, cluster size > 100). | | | | |

| Table S2. Regions showing GFA and NQA values differences between depressed patients with suicidal ideation (SI+), depressed patients without suicidal ideation (Depressed) and healthy controls (HC) by post hoc two-sample t- tests. | | | | |
| --- | --- | --- | --- | --- |
| Region | side | MNI coordinates (mm) | | |
|  |  | X | Y | Z |
| GFA value |  |  |  |  |
| SI+ < HC |  |  |  |  |
| Corpus callosum and anterior cingulate | L/R | ±12 | 32 | 2 |
| SI+ < Depressed |  |  |  |  |
| Corpus callosum and anterior cingulate | L | -14 | 41 | 6 |
|  |  |  |  |  |
| NQA value |  |  |  |  |
| SI+ < HC |  |  |  |  |
| Anterior cingulate | L/R | ±6 | 0 | 34 |
| SI+ < Depressed |  |  |  |  |
| Corpus callosum and anterior cingulate | L | -14 | 37 | 7 |
| All coordinates are given in Montreal Neurological Institute (MNI) space. (p < 0.05, cluster size > 100). | | | | |
